# Impaired wakefulness and rapid eye movement sleep in dopamine-deficient mice

**DOI:** 10.1186/s13041-021-00879-3

**Published:** 2021-11-18

**Authors:** Mitsuaki Kashiwagi, Mika Kanuka, Kaeko Tanaka, Masayo Fujita, Ayaka Nakai, Chika Tatsuzawa, Kazuto Kobayashi, Kazutaka Ikeda, Yu Hayashi

**Affiliations:** 1 International Institute for Integrative Sleep Medicine (WPI-IIIS), University of Tsukuba, Tsukuba, 305-8575 Japan; 2 Addictive Substance Project, Tokyo Metropolitan Institute of Medical Science, Setagaya-ku, Tokyo, 156-8506 Japan; 3 Doctoral Programs in Neuroscience, Degree Programs in Comprehensive Human Sciences, University of Tsukuba, Tsukuba, 305-8575 Japan; 4 Department of Molecular Genetics, Institute of Biomedical Sciences, Fukushima Medical University, Fukushima, 960-1295 Japan; 5 Department of Human Health Sciences, Graduate School of Medicine, Kyoto University, Kyoto, 606-8507 Japan

**Keywords:** Dopamine, Mouse, NREM sleep, REM sleep

## Abstract

Despite the established roles of the dopaminergic system in promoting arousal, the effects of loss of dopamine on the patterns of sleep and wakefulness remain elusive. Here, we examined the sleep architecture of dopamine-deficient (DD) mice, which were previously developed by global knockout of tyrosine hydroxylase and its specific rescue in noradrenergic and adrenergic neurons. We found that DD mice have reduced time spent in wakefulness. Unexpectedly, DD mice also exhibited a marked reduction in the time spent in rapid eye movement (REM) sleep. The electroencephalogram power spectrum of all vigilance states in DD mice were also affected. These results support the current understanding of the critical roles of the dopaminergic system in maintaining wakefulness and also implicate its previously unknown effects on REM sleep.

**Supplementary Information:**

The online version contains supplementary material available at 10.1186/s13041-021-00879-3.

## Introduction

In contrast to the rapidly growing knowledge on the anatomy of sleep–wake regulating circuits, the molecular mechanism of sleep–wake regulation remains largely unknown [1]. We focused on dopamine, a neurotransmitter that plays important roles in various brain functions including reward, motor control, and sleep–wake regulation. Psychostimulant drugs such as d-amphetamine and modafinil increase wakefulness [2]. Dopamine transporter knockout mice, in which extracellular dopamine is increased, have increased wakefulness [2]. Optogenetic or chemogenetic activation of dopaminergic neurons in the ventral tegmental area (VTA) [3, 4] or dorsal raphe [5] induces wakefulness, respectively. While the effect of activating the dopaminergic system seems well established, the effect of inactivation remains obscure. Lesion of dopaminergic ventral periaqueductal gray neurons with 6*-*hydroxydopamine reduces wakefulness [6]. Pharmacological inhibition [7] or genetic deletion [8] of the dopamine receptors also reduces wakefulness. By contrast, the effects of acute inhibition of VTA dopaminergic neurons using chemogenetics varied across studies, either reducing or having no effect on the time spent in wakefulness [3, 4]. Genetic ablation of VTA dopaminergic neurons also has apparently no effect [9]. In addition, the effect of inactivating the dopaminergic system on REM sleep remains elusive. For example, the effects of pharmacological [7] or genetic [8] intervention of dopamine receptors on REM sleep are diverse.

To directly address the effect of loss of dopamine on sleep and wakefulness, we focused on the DD mice [10]. DD mice were developed by global knockout of tyrosine hydroxylase plus its specific rescue in noradrenergic and adrenergic neurons using the dopamine beta hydroxylase promoter [10]. DD mice are hypoactive in their home cage [10]. However, their sleep architecture remained unknown. Thus, in the present study, we simultaneously recorded the electroencephalogram (EEG) and electromyogram (EMG) from DD mice to examine their sleep architecture (Additional file 1).

## Results

### DD mice exhibited impaired wakefulness and REM sleep

Dopamine-deficient (DD) mice were maintained with daily intraperitoneal administration of 50 mg/kg l-DOPA, starting from around postnatal day 10. Wildtype (WT) mice were administered with saline at least 3 times before EEG/EMG recording. EEG/EMG recordings were conducted 48 h after the last administration of 50 mg/kg l-DOPA (to DD mice) or saline (to WT mice). EEG/EMG signals were recorded for 24 h as previously described [11, 12].

**Fig. 1:**
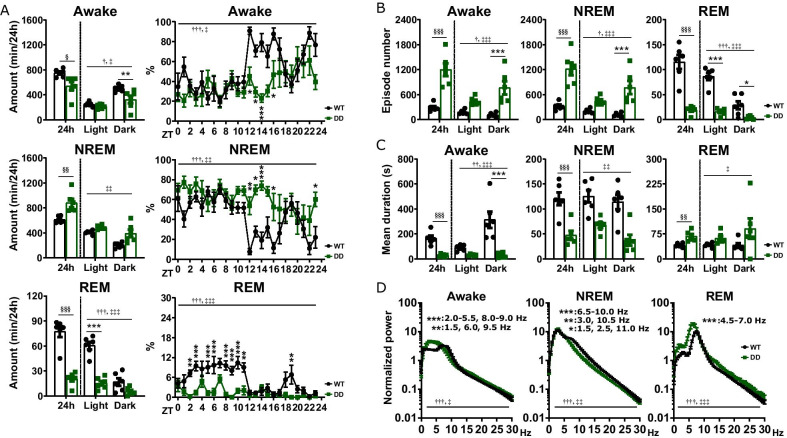
Analysis of sleep architecture of DD mice. **A** Time spent in wakefulness, NREM sleep, and REM sleep in WT mice and DD mice. *ZT* zeitgeber time. **B**, **C** Episode numbers and mean durations of each vigilance state in WT mice and DD mice. **D** EEG power spectrum of each vigilance state in WT mice and DD mice. Frequency ranges with significant difference in the Bonferroni’s test between WT mice and DD mice are described within each graph. Data represent mean ± SEM. n = 6 mice in each group. ^§^P < 0.05; ^§§^P < 0.005; ^§§§^P < 0.001 [unpaired t-test]; *^,†,‡^P < 0.05; **^,††,‡‡^P < 0.005; ***^,†††,‡‡‡^P < 0.001 [two-way repeated measures ANOVA followed by Bonferroni’s test; ^†^ and ^‡^ indicate significant main effect of intervention and significant interaction between intervention and time, respectively, in two-way repeated measures ANOVA, and * indicates significance in Bonferroni’s test]

DD mice exhibited reduced time spent in wakefulness and REM sleep, and increased time spent in non-REM (NREM) sleep (Fig. 1A). Wakefulness was reduced in the dark phase, whereas REM sleep was decreased in the light phase (Fig. 1A). For wakefulness and NREM sleep, DD mice exhibited reduced mean episode duration and increased episode number (Fig. 1B, C), demonstrating that these states are highly fragmented. Oppositely, for REM sleep, DD mice exhibited increased mean episode duration and reduced episode number (Fig. 1B, C). We also compared the EEG power spectrum of WT and DD mice. During wakefulness, DD mice exhibited increased EEG spectral power at 1.5–6.0 Hz and reduced power at 8.0–9.5 Hz (Fig. 1D). During NREM sleep, DD mice exhibited increased EEG spectral power at 2.5–3.0 Hz and reduced power at 1.5 Hz and 6.5–11.0 Hz (Fig. 1D). During REM sleep, DD mice exhibited increased EEG spectral power at 4.5–7.0 Hz (Fig. 1D). Thus, the EEG activity of DD mice was different from that of WT mice (Additional file 2).

## Discussion

In this study, we found abnormalities in the sleep architecture of DD mice. Wakefulness was reduced and highly fragmented, indicating that DD mice cannot properly maintain wakefulness. Our observations are in line with the previously reported hypoactive phenotype of DD mice [10] and the critical roles of the dopaminergic system in maintaining wakefulness [2–9]. Importantly, loss of VTA dopaminergic neurons did not affect the time spent in wakefulness according to a viral vector-mediated ablation study [9]. Thus, dopaminergic neurons in the other areas might contribute to maintaining wakefulness.

Unexpectedly, we also found that REM sleep is drastically reduced in DD mice. Considering that REM sleep typically follows deep NREM sleep, reduced REM sleep might be a result of fragmented NREM sleep in DD mice. However, it should be noted that dopamine D2 receptor knockout mice also exhibit highly fragmented NREM sleep in the dark phase and yet the amount of REM sleep is unaffected [3]. Genetic activation of dopaminergic neurons in the VTA or dorsal raphe does not increase the time spent in REM sleep [3–5]. On the other hand, dopaminergic neurons in the VTA are active during REM sleep according to fiber photometry recordings [3] and the dopamine concentration in the nucleus accumbens and prefrontal cortex, where the VTA dopaminergic neurons massively project, is increased during REM sleep according to measurements by microdialysis [13]. In addition, several drugs that are considered to antagonize dopamine receptors reduce the time spent in REM sleep [7]. Thus, dopamine might contribute to the positive regulation of REM sleep through unknown mechanisms. We also found abnormalities in the EEG power spectrum of DD mice, implying that dopamine also contributes to the patterns of neuronal synchrony and/or oscillation.

One technical limitation of this study is that there is a possibility that the daily l-DOPA treatment and/or consecutive intraperitoneal injections in DD mice might have had some effect on sleep. However, 50 mg/kg l-DOPA injection does not affect the amount of each vigilance state in WT mice [14]. In addition, at the timing of EEG/EMG recordings (48 h after the last 50 mg/kg l-DOPA injection), dopamine level in the striatum of DD mice is expected to be largely depleted according to our previous study (extracellular dopamine [fmol/10 min] 24 h after the last 50 mg/kg l-DOPA injection: WT 39.23 ± 3.76, DD mice 0.62 ± 0.12; 72 h after the last 50 mg/kg l-DOPA injection: WT 43.55 ± 4.80, undetectable in DD mice) [15]. Another technical issue is that the transgenic expression of tyrosine hydroxylase in DD mice might affect the level of other neurotransmitters such as noradrenaline, although we previously confirmed that noradrenaline level in the striatum of DD mice was unaffected (extracellular noradrenaline [fmol/10 min] 72 h after the last 50 mg/kg l-DOPA injection: WT 0.51 ± 0.07, DD 0.67 ± 0.09) [15].

In summary, by direct measurement of EEG/EMG and analysis of sleep architecture in DD mice, this study further supports the current understanding of the critical roles of dopaminergic system in maintaining wakefulness and implicates its overlooked effects on the positive regulation of REM sleep.

## Supplementary Information

**Additional file 1.** Materials and methods.

**Additional file 2.** Raw data presented in this study.

## Data Availability

All data are available upon reasonable request to the corresponding author.

## Abbreviations

REM sleep: Rapid eye movement sleep
NREM sleep: Non-REM sleep
DD mouse: Dopamine-deficient mouse
EEG: Electroencephalogram
EMG: Electromyogram
VTA: Ventral tegmental area
ZT: Zeitgeber time
