# Supplementary material for: Impaired wakefulness and rapid eye movement sleep in dopamine-deficient mice
Source: Mol Brain. 2021 Nov 18;14:170. doi: 10.1186/s13041-021-00879-3 (PMC8600805; doi:10.1186/s13041-021-00879-3)
Supplement: Supplementary file 1 — Additional file 1. Materials and methods. [file 13041_2021_879_MOESM1_ESM.doc]

Additional file 1

**Supplementary information**

**Materials and methods**

**Mice**

All animal experiments were approved by the institutional animal care and use committee of the University of Tsukuba. All animals were maintained according to the institutional guidelines of the Laboratory of Animal Resource Center, University of Tsukuba. Dopamine-deficient (DD) mice were maintained as previously described with daily intraperitoneal administration of 50 mg/kg L-DOPA (Nacalai Tesque, Kyoto, Japan) dissolved in 2.5 mg/ml ascorbic acid (Nacalai Tesque) solution in saline [1]. Male and female wildtype (WT) and DD mice were used in this study. Mice were group housed after weaning (2-5 mice/cage) and single housed after surgery under a 12-h light/dark cycle. Food and water were given at libitum.

**EEG/EMG recording**

EEG/EMG recordings were conducted as previously described [2,3] with slight modifications. Mice were anesthetized with isoflurane and placed in a stereotaxic apparatus (David KOPF Instruments, Tujunga, USA). The height of bregma and lambda was adjusted to be within a difference of 0.1 mm and EEG (electroencephalogram) and EMG (electromyogram) electrodes were implanted. EEG electrodes (stainless steel recording screws) were implanted epidurally over the parietal area (1 mm anterior to lambda, 1 mm lateral to midline) and the cerebellar area. EMG electrodes (stainless steel wires) were bilaterally implanted into the neck muscle.

Mice were allowed to recover for at least 2 weeks after surgery. The mice were then moved to a sleep recording chamber and connected to an amplifier for EEG/EMG recording. Mice were acclimatized to the sleep recording chamber for 1 week. To control for the effects of intraperitoneal injection, WT mice were administered with 0.2 ml of saline at least 3 times before EEG/EMG recording. EEG/EMG recordings were conducted 48 h after the last administration of 50 mg/kg L-DOPA (to DD mice) or saline (to WT mice). EEG/EMG signals were recorded for 24 h.

**EEG/EMG analysis**

EEG/EMG signals were filtered (band pass 0.5-250 Hz) and collected and digitized at a sampling rate of 512 Hz via VitalRecorder (Kissei Comtec, Matsumoto, Japan). EEG signals were subjected to fast Fourier transform and further analysis using SleepSign (Kissei Comtec). The vigilance state in each epoch was manually classified as REM sleep, NREM sleep, or wake based on absolute delta (0.5-4 Hz) power, theta (6-10 Hz) power to delta power ratio, and the integral of EMG signals. We applied 4-s epochs throughout the study. If a single epoch contained multiple states, the state with the highest occupancy was assigned.

**Quantification and statistical analysis**

The statistical details of the experiments are provided in the figure legends. Sample size was determined based on previous relevant studies. Each graph represents mean ± SEM. Data were analyzed with Prism (Graph Pad, San Diego, USA). A p-value of less than 0.05 was considered as statistically significant.

**References**

1. Nishii, K., Matsushita, N., Sawada, H., Sano, H., Noda, Y., Mamiya, T., Nabeshima, T., Nagatsu, I., Hata, T., Kiuchi, K., *et al.* (1998). Motor and learning dysfunction during postnatal development in mice defective in dopamine neuronal transmission. J. Neurosci. Res *54*.

2. Hayashi, Y., Kashiwagi, M., Yasuda, K., Ando, R., Kanuka, M., Sakai, K., and Itohara, S. (2015). Cells of a common developmental origin regulate REM / non-REM sleep and wakefulness in mice. Science (80-. ). *350*, 957–961.

3. Kashiwagi, M., Kanuka, M., Tatsuzawa, C., Suzuki, H., Morita, M., Tanaka, K., Kawano, T., Shin, J.W., Suzuki, H., Itohara, S., *et al.* (2020). Widely distributed neurotensinergic neurons in the brainstem regulate NREM sleep in mice. Curr. Biol. *30*, 1002-1010.e4.
